# Anticandida and antibiofilm activities of extract from *Schinopsis brasiliensis* Engl. against *Candida* spp.

**DOI:** 10.1590/1807-3107bor-2024.vol38.0016

**Published:** 2024-03-11

**Authors:** Vanessa de Carvalho JOVITO, Jefferson Muniz de LIMA, Marianne de Lucena RANGEL, Brenna Louise Cavalcanti GONDIM, Paula Lima NOGUEIRA, Ana Claúdia Dantas de MEDEIROS, Marianna Vieira SOBRAL, Ricardo Dias de CASTRO, Lúcio Roberto Cançado CASTELLANO

**Affiliations:** (a) Universidade Federal da Paraíba – UFPB, Graduate Program in Dentistry, Departament of Clinical and Social Dentistry, João Pessoa-PB, Brazil.; (b) Universidade Estadual da Paraíba – UEPB, Departament of Pharmaceutical Sciences, Campina Grande, PB, Brazil.; (c) Universidade Federal da Paraíba – UFPB, Departament of Pharmaceutical Sciences, João Pessoa, PB, Brazil.

**Keywords:** Pharmacology, Phytotherapy, Microbiology, Biofilms, Candidiasis, Oral

## Abstract

The pathogenic nature of infections caused by Candida spp. underscores the necessity for novel therapeutic agents. Extracts of *Schinopsis brasilienses* Engl are \ a promising source of agents with antifungal effects. This study aimed to assess the antifungal potential of the leaf extract of *S. brasilienses*. The antifungal activity was evaluated by determining the minimum inhibitory concentrations and fungicide concentrations (MIC and MFC). The antibiofilm potential was assessed by counting colony-forming units/mL. The study examined the inhibition kinetics of fungal growth and potential synergism between gallic acid or the extract and nystatin using the Checkerboard method. Cytotoxicity was evaluated through the MTT assay. The extract exhibited antifungal effect against all tested strains, with MIC and MFC ranging from 31.25–250 μg/mL. Gallic acid, the main isolated compound, displayed a MIC of 2000 μg/mL. The extract of *S. brasilienses* at 31.25 μg/mL inhibited the formation of biofilm by *C. albicans* and significantly reduced the mass of mature biofilm after 24 and 48 h (p < 0. 05). At a concentration of 125 μg/mL, the extract demonstrated significant inhibition of fungal growth after 6 hours. The combination of gallic acid or extract with nystatin did not exhibit synergistic or antagonistic effect. Furthermore, the extract did not induce cytotoxicity to a human cell line. The extract of *S. brasiliensis* demonstrates antifungal activity against *Candida*, generally exhibiting fungicidal action and capacity to inhibit biofilm formation as well as reduce mature biofilms. Additionally, the extract showed low cytotoxicity to human cells.

## Introduction

Brazil is a country with a vast diversity of plants, many of which have sparked scientific interest due to their biological effects on population health. Approximately 80% of developing countries utilize alternative methods to address their pathologies.^1,2^

Medicinal plants have proven to be an easily accessible and cost-effective alternative that is gaining traction alongside conventional treatment methods in the market. Conventional treatments are often associated with high costs and undesirable effects such as toxicity, microbial resistance, and antagonistic interactions with other drugs.^3,4^

Schinopsis brasiliensis Engl. is a plant found in the Brazilian semiarid and is known as “baraúna”. It has been traditionally used in the treatment of certain conditions. Gallic acid (3,4,5-trihydroxybenzoic acid) is the chemical marker in this plant and is an important polyphenol known for its antioxidant, anti-inflammatory, and antimicrobial properties.^5,6^

This information supports the hypothesis that extracts derived from *S. brasiliensis* have an effect against microorganisms, including those associated with fungal infections, such *Candida* spp., which are opportunistic pathogens commonly found in immunocompromised patients and are responsible for high morbidity and mortality rates, as well as increased healthcare costs.^7^

New therapeutic agents are needed for the treatment of infections by *Candida* spp., which can adapt to the environment and exhibit the ability to form biofilms. Considering that extracts obtained from *S. brasiliensis* are potential sources of chemical agents with an antifungal effect,^8-11^ the objective of this work was to evaluate the anti-*Candida* potential of the leaf-rotavaporated extract of Schinopsis brasilienses Engl.

## Methodology

### Plant extract

The leaves of *S. brasiliensis* were collected in the region of Campina Grande, Paraíba, Brazil (7 ° 13 50 S, 35 ° 52 52 W), respecting the time and period of ideal collection. A sample was prepared and deposited at the herbarium Professor Jayme Coelho de Morais (Herbarium Code EAN- 14049) of the Federal University of Paraíba under the number EAN-14049. The plant material was dried in an oven at 40 ± 1°C with air circulation and ground in a mill with a particle size of 10 mesh. The powder of the ground plant (100 g) was extracted by percolation exhaustively with 96% ethanol, and subsequently the concentration was obtained on a rotary evaporator. The study was registered in the National System of Genetic Heritage Management and Associated Traditional Knowledge - SISGEN (no. A4ABDFD).

### Microorganisms

The following *Candida* strains were obtained from the American Type Culture Collection (ATCC): *Candida* albicans ATCC 60193, *Candida kruse*i ATCC 34135, *Candida tropicalis* ATCC 750; from the Centralbureau voor Schimmelcultures (CBS): Candida albicans CBS 562, *Candida tropicalis* CBS 94; and from the Zimotécnico Institute, Banco de Luiz de Queiroz of Unicamp - Campinas, SP, Brazil: Candida glabrata IZ 07 and Clinical strain of Candida albicans (CAM) isolated from the oral cavity and provided by the Clinical Mycology Laboratory of the Department of Pharmaceutical Sciences of the UFPB.

### Determination of the minimum inhibitory concentration (MIC) and minimum fungicide concentration (MFC)

The MIC is defined as the lowest possible concentration that inhibits the growth of the fungal strain, being determined using the microdilution technique described by the Clinical and Laboratory Standards Institute.^12^The yeast suspension was prepared in sabouraud dextrose broth (SDB) (Kasvi, Curitiba, Brasil) and adjusted to turbidity equivalent to 2.5 10^3^ CFU/mL, 530 nm, abs 0.08-0.13.

For microdilution, sterile 96-well flat-bottomed microdilution plates (Cellstar^®^) were used, which initially received 100 µL of SDB. Subsequently, 100 µL of the substances under study were inserted into the first well of each column, followed by the serial microdilution process, providing concentrations ranging from 2000 to 15.62 µg/mL. Subsequently, 100 µL of the inoculum of the fungal strains were added to each well.

Nystatin (Sigma-Aldrich, St. Louis, MO, USA) was used as a positive control. Control of strain viability and culture medium sterility was also carried out. The plates were incubated for 24 h at 35°C. Three independent experiments were performed in triplicate. Cell aggregates at the bottom of the wells were observed and confirmed using the dye 2,3,5-triphenyl tetrazolic chloride (TCT) (Dinâmica, Brazil); 50 μL were added to the wells and the plates were incubated again for 24 h at 35°C. The reading was confirmed by the presence of red-stained viable microorganisms in the wells.^13^

The MFC is defined as the lowest possible concentration that inhibits the growth of the fungal strain on solid media. Fifty microliter aliquots corresponding to the MIC and two multiple concentrations of this concentration were subcultured on sabouraud dextrose agar (SDA) (Kasvi, Curitiba, Brazil). These plates were incubated for 24 h at 35ºC; results were determined by visual observation of fungal growth in the culture medium. The MFC/MIC ratio was calculated to determine whether the substance had a fungistatic (MFC/CIM > 4) or fungicidal activity (MFC/MIC < 4).^14^

### Kinetics of fungal growth inhibition

The study of the interference of the extract in the growth and multiplication of fungal cells of *C. albicans* ATCC 60193 was carried out by counting the colony-forming units (CFU) based on previous studies.^15,16^ The evaluation times defined for this test were T0 (initial), T1 (1 hour after onset), T2 (2 hours), T6 (6 hours), T8 (8 hours), T12 (12 hours), and T24 (24 h after the start of the assay).

The assay was performed in a 96-well plate using the same protocol as the microdilution technique^12^ with the extract at MIC, MIC x 2, and MIC x 4. Nystatin was used as the positive control. Growth control of the tested strain and control of sterility of the culture medium was performed in parallel.

For evaluation of fungal growth inhibition kinetics, 10 μL of well contents after homogenization were seeded onto Petri dishes containing SDA at the predefined time intervals and incubated at 35°C for 24 h for subsequent CFU count. After incubation, the CFU were counted and the log_10_-transformed CFU/mL was plotted as a fungal cell death curve.

### Evaluation of the anti-biofilm activity of the extract

The anti-biofilm activity of the products were evaluated at three different times: biofilm formation and 24- and 48-hour reduction of mature Candida biofilm.

The tests were performed in triplicate on *C. albicans* ATCC 60193 and C. tropicalis ATCC 750 biofilms and on multispecies (*C. albicans* + C. tropicalis) biofilm. Nystatin was used as a control in all groups. Growth control of the tested strain and control of culture medium sterility were performed in parallel.

### Evaluation of anti-biofilm formation

One hundred microliters of the inoculum prepared in RPMI plus 2% sucrose containing 2.5 x 10^5^ CFU/mL were transferred to each well of a flat bottom 96-well microdilution plate containing 100 μL of RPMI (Roswell Park Memorial Institute medium, Sigma, Germany) with the aid of a pipette. Then, 100 μL of the extract at different concentrations was added to the corresponding wells. The plate was incubated for 48 h at 35°C, allowing the yeast to remain adhered to the bottom of the wells.

To perform the reading and quantification of the formed biofilm, after the incubation time, the wells were washed twice with 200 μL of phosphate buffered saline solution (PBS) and air-dried for 45 min. In each well was added 100 μL of 0.4% aqueous crystal violet solution, which remained in contact with the biofilm for 45 min. After incorporation of the dye, the wells were washed three times with 200 μL of sterile distilled water and immediately bleached with 200 μL of 95% ethanol. After 45 minutes of the latter procedure, 100 μL of the bleached solution were transferred to a well of a new plate and the amount of violet crystal was measured at 600 nm in an absorbance reader (GloMax-Multi, Promega-USA).^17^

The absorbance values obtained in the wells of the tested concentrations and growth control were used to calculate the percentage of inhibition (% inhibition) of biofilm formation due to the action of the substance.

### Evaluation of mature biofilm reduction

One hundred microliters of the RPMI prepared inoculum plus 2% sucrose containing 2.5 x 10^5^ CFU/mL were transferred to each well of a 96-well flat bottom microdilution plate containing 100 μL RPMI with the aid of a pipette and incubated in an oven at 35°C for 48 hours to form the mature biofilm.

Then, the culture medium was aspirated from the wells to remove planktonic cells. Wells were washed twice with 200 μL of PBS. After washing, 100 μL of the RPMI medium was transferred to each well. Then, 100 μL of the extract at the concentrations tested were added to the wells and staining of the biofilm was performed for a period of 24 and 48 hours

Controls and quantification procedures of the formed biofilm were performed in the same manner as described in the previous assay.

### Synergism evaluation - Checkerboard method

The combined effect of the two substances (nystatin with leaf extract of *S. brasiliensis* and nystatin with Gallic acid) was assessed with the microdilution - checkerboard technique for derivation of the fractional inhibitory concentration index (FIC Index).

The turbidity of the fungal suspensions was adjusted in a spectrophotometer at a concentration of 10^5^ CFU/mL. MIC solutions of the tested products were used. Initially, 100 μL of the culture medium was added to the wells of a 96-well U-bottomed microplate (Cellstar^®^). Then, 50 μL of each tested product at various concentrations (MIC÷8, MIC÷4, MIC÷2, MIC, MICx2, MICx4 and MICx8) were added vertically (nystatin) and horizontally (extract) to the microplate. Finally, the culture medium was inoculated with 10 μL of the *C. albicans* fungal suspension. Fungal growth was evidenced by the use of the TCT dye. The assay was performed in triplicate, and the microplates were incubated at 35°C for 48 hours.^18,19^

The FIC index was calculated by adding FIC^A^ + FIC^B^, where A is the extract and B is nystatin. The FIC^A^ was calculated by the ratio MIC^A^ / MIC^A^ alone, while the FIC^B^ was calculated as MIC^B^ / MIC^B^ alone. This index was interpreted as follows: synergism (< 0.5), additivity (0.5–1.0), indifference (> 1 and < 4) or antagonism (> 4.0).

### Cytotoxicity assay

MTT assay was performed to evaluate the cytotoxicity of the extract *S. brasiliensis* against HEK293 cells, which is a human embryonic kidney cell line. HEK293 cells were cultured in Dulbecco’s Modified Eagle’s Medium (DMEM) supplemented with glucose, 10% fetal bovine serum, 100 U/mL penicillin, and 100 μg/mL streptomycin at 37°C in a humidified atmosphere with 5% CO_2_. Cells (3 × 10^5^ cells/mL) were seeded onto 96-well plates and incubated with the *S. brasiliensis* extract (3.9–500 µg/mL) dissolved in DMSO, at concentrations not exceeding 0.25%. The positive control was treated with 20% DMSO. Three independent experiments were performed in quadruplicate. After culturing for 72 h, the supernatant was discarded, and the 3-(4,5-dimethylthiazol-2-yl)-2,5-diphenyltetrazolium bromide (MTT) solution (5 mg/mL) was added and incubated for another 4 h. The deposited formazan was dissolved with sodium dodecyl sulfate (SDS) (100 μL) (MOSMANN, 1983).^20^ The optical densities were measured using a microplate reader (Synergy HT, BioTek).

### Statistical analyses

The data were analyzed by paired T-test, ANOVA with Tukey’s post hoc test, Mann-Whitney, and Kruskal-Wallis with alpha error of 5%.

## Results

### MIC and MFC

The extract from the leaves of *S. brasiliensis* showed antifungal effect on all the strains tested, with MIC and MFC values varying between 31.25 and 250 μg/mL (Table 1), with the strains of *C. albicans* (ATCC 60193 and CBS 562) having the lowest MIC and MFC values. Nystatin was used as a control and was tested at concentrations ranging from 12 to 0.18 μg/mL

**Table 1:** MIC and MFC of of *S. brasiliensis* extract against *Candida* spp.

Strains	S. brasiliensis			Nystatin		
	MIC	MFC	MFC/MIC	MIC	MFC	MFC/MIC
	
	µg/mL	µg/mL		µg/mL	µg/mL	
*C. albicans*	31.25	125	4 (Fungistatic)	0.375	0.375	1 (Fungicide)
ATCC 60193						
*C. krusei*	125	125	1 (Fungicide)	3	3	1 (Fungicide)
ATCC 34135						
*C. tropicalis*	250	250	1 (Fungicide)	1.5	1.5	1 (Fungicide)
ATCC 750						
*C. albicans*	31.25	31.25	1 (Fungicide)	0.375	0.75	2 (Fungicide)
CBS 562						
*C. tropicalis*	250	250	1 (Fungicide)	1.5	1.5	1 (Fungicide)
CBS 94						
*C. albicans* CAM	250	250	1 (Fungicide)	0.375	0.75	2 (Fungicide)

Concerning the MFC/MIC ratio, the leaf extract of *S. brasiliensis* showed fungicide activity for all Candida species analyzed, except for *C. albicans* ATCC 60193 which was shown to be fungistatic.^14^

Gallic acid, which has high antioxidant and antimicrobial potential^8^, showed activity only for two strains (*C. albicans* ATCC 60193 and C. glabrata IZ 07) when analyzed at the same concentration of *S. brasiliensis* extract that was 2000 μg/mL, considering that the antifungal activity can be attributed to another chemical component in extract composition (Table 2).

**Table 2:** MIC of gallic acid against *Candida* spp.

Cepas	CIM

	(µg/mL)
*C. krusei* ATCC 34135	+
*C. tropicalis* ATCC 750	+
*C. albicans* ATCC 60193	2,000
*C. glabrata* IZ 07	2,000

+ Fungal growth

### Effect of the *S. brasiliensis* extract on the growth kinetics of *C. albicans*

**Figure 1:**
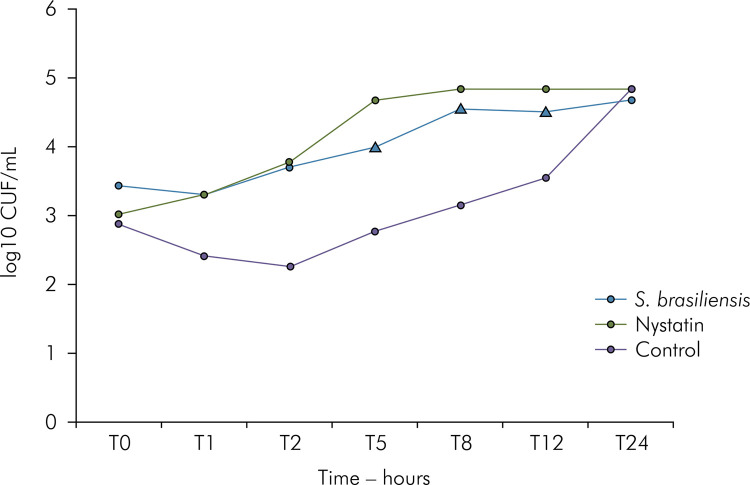
Kinetics test showing the behavior of the *S. brasiliensis* extract, nystatin, and control at the MICx4 concentration during 24 hours (*p < 0.05).

At the MIC and MICx2, the *S. brasiliensis* extract did not significantly reduce the number of *C. albicans* ATCC 60193 CFUs at the times evaluated compared to the control. However, at the concentration of 125 μg/mL, the extract evaluated significantly reduced the growth after 6 hours of incubation (p<0.05). Nystatin significantly reduced fungal growth (p < 0.05) from the first hour of incubation, and this effect was prolonged for up to 12 hours (Figure 1).

### Evaluation of the anti-biofilm activity of the leaf extract of *S. brasiliensis*

*C. albicans* ATCC 60193 biofilm was significantly reduced in the groups of *S. brasiliensis* extract compared with nystatin (p < 0.05, Mann-Whitney Test). Statistically significant differences (p < 0.05, Kruskal-Wallis test) were found for CIMx8 (250 μg/mL) of the extract in groups G1 and G3 and for MICx4 (125 μg/mL) and MICx8 (250 μg/mL) for the G2 group (Table 3).

**Table 3:** Effect of *S. brasiliensis* extract on the inhibition of biofilm formation - G1- and reduction of mature biofilm 24h in contact with extract - G2 - and 48hrs in contact with extract - G3 - of the mature biofilm of *C. albicans* ATCC 60193. Values are expressed as percentage (%).

Concentration	G1		G2		G3	

	*S. brasiliensis*		*S. brasiliensis*	Nystatin	*S. brasiliensis*	Nystatin
CIM	42%^Aa^	no inhibition^Ba^	76%^Aa^	no inhibition^Ba^	55%^Aa^	10%^Ba^
2 CIM	73%^Aa^	no inhibition^Ba^	86%^Aa^	no inhibition^Ba^	68%^Aa^	35%^Ba^
4 CIM	63%^Aa^	no inhibition^Ba^	100%^Ab^	6%^Ba^	85%^Aa^	41%^Bb^
8 CIM	100%^Ab^	no inhibition^Ba^	100%^Ab^	no inhibition^Ba^	100%^Ab^	24%^Ba^

Different upper-case letters in lines represent statistically significant differences (Mann-Whitney Test, p < 0.05) between substances (leaf extract of *S. brasiliensis* and nystatin) in each group and at the same concentration; Different lowercase letters in each column represent statistically significant differences (Kruskal-Wallis test, p<0.05) between different concentrations of the same substance.

The C. tropicalis ATCC 750 biofilm was significantly reduced in the extract compared to nystatin in groups G1 and G2 with p < 0.05. MICx2 (500 μg/mL) and MICx4 (1000 μg/mL) were significantly different (p < 0.05) than MICx8 (2000 μg/mL) in G1 and G2 (Table 4).

**Table 4:** Effect of *S. brasiliensis* extract on the inhibition of biofilm formation - G1 - and reduction of mature biofilm 24h in contact with extract - G2 - and 48hrs in contact with the G3 extract of the mature biofilm of *C. tropicalis* ATCC 750. Values expressed in percentage (%).

Concentration	G1		G2		G3	

	*S. brasiliensis*	Nystatin	*S. brasiliensis*	Nystatin	*S. brasiliensis*	Nystatin
CIM	no inhibition^Aa^	47%^Ba^	64%^Aa^	21.22%^Aa^	78%^Aa^	2%^Ba^
2 CIM	no inhibition^Aa^	34%^Ba^	66%^Aa^	32.84%^Aa^	89%^Aa^	23%^Bb^
4 CIM	23%^Aa^	40%^Ba^	80%^Aa^	21.69%^Aa^	82%^Aa^	21%^Bb^
8 CIM	100%^Ab^	44%^Ba^	84,59%^Ab^	22.09%^Aa^	99%^Ab^	17%^Bb^

Different upper-case letters in the lines represent statistically significant differences (Mann-Whitney Test, p < 0.05) between substances (Extract of leaf of *S. brasiliensis* and nystatin) in each group and at the same concentration; Different lowercase letters in each column represent statistically significant differences (Kruskal-Wallis test, p < 0.05) between different concentrations of the same substance.

In the multispecies biofilm, the reduction was different between the extract and nystatin in G1 and G3, and between the extracts concentrations there was a difference in the MICx8 between G1 and G3, both with p < 0.05 (Table 5).

**Table 5:** Effect of extract of *S. brasiliensis* on the inhibition of biofilm formation - G1 and reduction of biofilm 24h in contact with extract - G2 and 48hrs in contact with the G3 extract of the mature multispecies biofilm (*C. albicans* ATCC 60193 + *C. tropicalis* ATCC 750). Values are expressed in percentage (%).

Concentration	G1		G2		G3	

	*S. brasiliensis*	Nystatin	*S. brasiliensis*	Nystatin	*S. brasiliensis*	Nystatin
MIC	63%^Aa^	38%^Ba^	39%^Aa^	79%^Ba^	76%^Aa^	3%^Ba^
2 MIC	73%^Aa^	14%^Bb^	47%^Aa^	76%^Aa^	88%^Aa^	13%^Bb^
4 MIC	89%^Aa^	22%^Bb^	56%^Aa^	81%^Bb^	92%^Aa^	27%^Bb^
8 MIC	100%^Ab^	26%^Bb^	72%^Aa^	69%^Aa^	96%^Ab^	25%^Bb^

Different upper-case letters in lines represent statistically significant differences (Mann-Whitney Test, p < 0.05) between substances (leaf extract of *S. brasiliensis* and nystatin) in each group and at the same concentration; Different lowercase letters in each column represent statistically significant differences (Kruskal-Wallis test, p < 0.05) between different concentrations of the same substance.

### Synergism evaluation - Checkerboard method

The FIC of the *S. brasiliensis* extract in association with nystatin was the same as the FIC of gallic acid in combination with nystatin: 1.125 (interpreted as indifferent, according to the methodology adopted).

### Cytotoxicity

**Figure 2:**
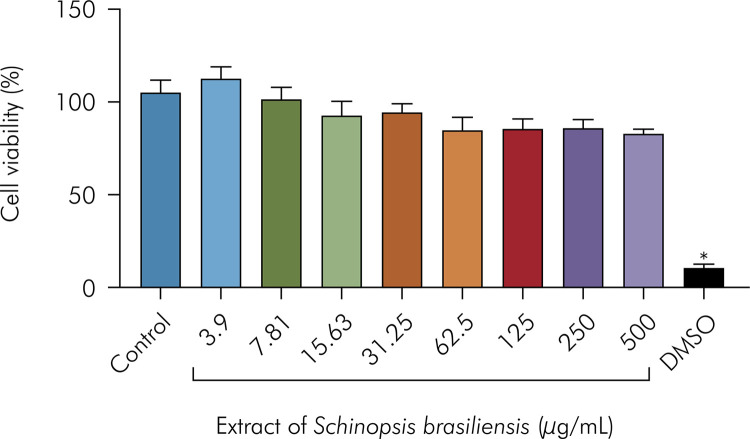
Cytotoxicity of the *S. brasiliensis* extract against HEK-293 cell line for 72 hours using the MTT assay. Data are presented as the mean ± SEM of three independent experiments, tested in different concentrations (3.9–500 µg/mL), in quadruplicate. DMSO at 20% was used as a positive control. *p <0.05 *vs* control analyzed by one-way analysis of variance (ANOVA) followed by Tukey’s post-test.

Figure 2 shows that the *S. brasiliensis* extract did not induce a significant cytotoxicity against a human embryonic kidney cell line, HEK293 cells, which are among the most commonly used cell lines in the study of drug toxicity. The tested concentrations (3.9–500 µg/mL) did not reduce the number of viable cells, showing values similar to the cell growth control.

## Discussion

The antimicrobial activity of the leaf of *S. brasiliensis* was analyzed against *C. albicans* (10231) strain and some bacterial strains, showing antifungal activity.^21^ Similarly, antifungal activity was also found for all strains of Candida spp. analyzed, with the effect of *C. albicans* showing better results, with MIC of 31.25 μg/mL.

Plant extracts with MIC values between 100 and 500 μg/mL are considered promising for possible clinical use.^22^ In this study, the MIC values observed reinforce the potential of the leaf extract of *S. brasiliensis* for treatment of fungal infections.

The largest component in the *S. brasiliensis* extract is gallic acid, a phenolic compound of plants with antioxidant, anti-inflammatory, and antimicrobial activity.^5^ In this study, gallic acid at a concentration equivalent to the extract of *S. brasiliensis* showed antifungal activity only to two fungal strains, with a higher MIC than the MIC of the extract.

In general, the antifungal activity is attributed to major compounds in the extracts. When these compounds are evaluated alone, they may show weaker activity and a limited spectrum of action compared to the extracts. This can be explained by the presence of other compounds in the extract, which also participate in the therapeutic effect and when associated with gallic acid, form a phytocomplex. Among compounds that may be acting synergistically are the secondary metabolites present in plants such as rutin, quercetin, and caffeic acid in low concentrations. The antimicrobial activity may also be related to the presence of other active substances in the extract such as flavonoids and tannins.^23^

The dental biofilm is defined as a complex community of microorganisms surrounded by an extracellular matrix of polysaccharides, adhered to one another on a surface or interface. Most of the persistent infections associated with dental biofilm are caused by fungi of the Candida genus.^24^

Studies on anti-biofilm activities of herbal extracts and essential oils have been reported in the literature,^25,26^ but this is the first study to use *S. brasiliensis* against biofilms of opportunistic pathogens. Candida spp. have important characteristics in the adhesion to this biofilm, which serves as protective deposits of microorganisms, which makes them more resistant to the activity of antimicrobial agents.^27,28^ Therefore, the early interruption of the formation and maturation of this biofilm is very important.^26^ The extract of *S. brasiliensis* significantly reduced biofilm formation and maturation compared to nystatin at the same concentrations.

In a previous study, the effective concentration of antimicrobial agents was found to be 10 to 1000 times higher than MIC in conventional liquid media in planktonic cells.^29^

The *S. brasiliensis* extract at concentrations of 31.25 and 62.5 μg/mL (MIC and MICx2) caused no death of *C. albicans* ATCC 60193 during a 24-hour period. However, at the concentration of 125 μg/mL, the extract was significantly more effective than the growth control after 6 hours of exposure. No study was found in the literature that evaluated the kinetics of the leaf extract of *S. brasiliensis*, which reinforces the innovative character of this work.

Due to the antifungal potential of the *S. brasiliensis* extract, the interaction between the extract and the gallic acid and nystatin through the FIC Index. The results showed that the association is not necessary because the effects of both the extract and the gallic acid were indifferent when associated with nystatin. No studies were found in the literature that relate the association of this extract with antifungals.

Because medicinal plants may have substances that are toxic to cells, cytotoxicity of the extract was analyzed.^30^ Our findings indicate that the extract of *S. brasiliensis* at concentrations capable of inhibiting fungal growth did not cause a reduction in the number of viable HEK293 cells, a human cell line. The toxicity of the shell extract of *S. brasiliensis* was assessed in Artemia salina, and a LC_50_ (median lethal concentration) of 428 μg/mL was found.^31^Acute toxicity of the leaf extract at a concentration of 2000 mg/mL was evaluated in mice, and no organ damage or death was observed, establishing the low toxicity of the extract.^23,24^ Other authors also evaluated the toxicity of the shell extract in mice and found similar studies.^31^

Toxicity studies are fundamental in the discovery and development of new active compounds for the treatment of diseases. The toxicity of gallic acid was evaluated in silico and a 2000 mg/kg was found as LD_50_ (median lethal dose), indicating the low toxicity of the compound.^32^ A literature review shows that the toxicity of the *S. brasiliensis* extract was analyzed in different experimental models. In vitro studies evaluated the cytotoxicity against fibroblast cell lines and found an LC_50_ of 6.14 mg/mL and IC_50_ (median inhibitory concentration) that ranged from 49.53 to 82 μg/mL. In in vivo studies, models of Artemia salina and Ceriodaphnia dubia were tested and obtained LC_50_ ranging from 962.97 µg/mL to 1.91 mg/mL.^33^

The ethanolic extract and fractions of *S. brasiliensis* Engl. (hexane fraction, chloroform fraction, and ethyl acetate fraction), with gallic acid as the main compound of the samples, were evaluated using IC_50_ for their potential to cause hemolysis in human red blood cells, and demonstrated low cytotoxicity of the concentrations tested in all samples, with IC_50_ ranging from 15.40 to 50.27 mg/mL. The selectivity index (SI), which indicates how many times more the substance is effective against specific target cells than human cells has also been determined. From the classification used, the SI of all tested samples was superior to the parameter, indicating its high selectivity, and was more toxic to microorganisms and less toxic to human cells.^34^

There is a diversity of researches in the field of natural products, and it is very difficult to compare studies, because of different methodologies, plant parts used, antimicrobial agents, among others. In addition, few studies are available in the literature on *S. brasiliensis* and its use in dentistry. Therefore, this study provides new knowledge and suggests that new studies be conducted on immunomodulation, tests in vivo, and development of products for the treatment of oral candidiasis.

## Conclusion

The leaf extract of *S. brasiliensis* had an antifungal activity on strains of the genus Candida, presenting mostly fungicidal action, especially after 6 hours of exposure. It also presented the ability to inhibit the formation and reduce maturation of Candida spp. (uni and multispecies) biofilm; the association with nystatin did not affect the activity. The tested extract did not exhibit cytotoxicity to a human cell line at concentrations with antifungal activity. The results suggest that the extract tested is promising in the development of products for oral candidiasis treatment.
